# Dynamic Characterization of Protein and Posttranslational Modification Levels in Mycobacterial Cholesterol Catabolism

**DOI:** 10.1128/mSystems.00424-19

**Published:** 2020-01-07

**Authors:** Jun-Yu Xu, Lei Zhao, Ying Xu, Bolin Li, Linhui Zhai, Minjia Tan, Bang-Ce Ye

**Affiliations:** a Laboratory of Biosystems and Microanalysis, State Key Laboratory of Bioreactor Engineering, East China University of Science and Technology, Shanghai, China; b State Key Laboratory of Drug Research, Shanghai Institute of Materia Medica, Chinese Academy of Sciences, Shanghai, China; Princeton University

**Keywords:** quantitative acetylome, quantitative propionylome, quantitative phosphorylome, quantitative proteome, cholesterol catabolism, mycobacteria

## Abstract

Cholesterol of the host macrophage membrane is vital for mycobacterial infection, replication, and persistence. During chronic infection within host lung tissues, cholesterol facilitates the phagocytosis of mycobacteria into macrophages. Cholesterol degradation leads to increased ﬂux of acetyl-coenzyme A (CoA) and propionyl-CoA, providing energy and building blocks for virulence macromolecules as well as donors for global protein acylation. Potential functions of lysine acylation are gradually revealed in bacterial survival and pathogenesis. However, the mycobacterial proteome and posttranslational modification (PTM) changes involved in the cholesterol catabolism bioprocess remain unclear. Here, we used nonpathogenic Mycobacterium smegmatis as a model and simultaneously monitored mycobacterial proteome and acetylome changes in the presence of glucose and cholesterol. We discovered that cholesterol metabolic enzymes were upregulated with respect to both protein expression levels and lysine acylation levels during the metabolic shift from glucose to cholesterol. After that, adenylating enzymes related to cholesterol metabolism were proven to be precisely regulated at the propionylation level by mycobacterial acyltransferase M. smegmatis Kat (*Ms*Kat) in response to cellular propionyl-CoA accumulation. Furthermore, the kinase expression and phosphorylation levels were also changed along with fluctuations in cholesterol levels. Our results expanded current knowledge of acylation regulation in the cholesterol catabolism of mycobacteria and provided references for possible antimycobacterium strategy.

**IMPORTANCE** Cholesterol assimilation is a critical step in mycobacterial chronic infection. However, knowledge from the dynamic characterization of cholesterol metabolism in mycobacteria at the protein expression and PTM levels remains limited. Our study uncovered the landscape of protein expression, lysine acetylation, lysine propionylation, and S/T/Y phosphorylation during the metabolic changes from glucose to cholesterol in mycobacteria. The data showed that cholesterol-induced carbon shift resulted in the elevation of protein expression and lysine acylation in diverse metabolic enzymes involved in cholesterol degradation and that the presence of cholesterol also promoted the perturbations at the phosphorylation level in the kinase system in mycobacteria. This study systematically characterized the regulation of cholesterol catabolism at several different levels, which provided the detailed references in mycobacterial proteome and potential antimycobacterial strategies.

## Abstract (precis)

Cholesterol assimilation is a critical step in mycobacterial chronic infection. However, knowledge from the dynamic characterization of cholesterol metabolism in mycobacteria at the protein expression and PTM levels remains limited. Our study uncovered the landscape of protein expression, lysine acetylation, lysine propionylation, and S/T/Y phosphorylation during the metabolic changes from glucose to cholesterol in mycobacteria. The data showed that cholesterol-induced carbon shift resulted in the elevation of protein expression and lysine acylation in diverse metabolic enzymes involved in cholesterol degradation and that the presence of cholesterol also promoted the perturbations at the phosphorylation level in the kinase system in mycobacteria. This study systematically characterized the regulation of cholesterol catabolism at several different levels, which provided the detailed references in mycobacterial proteome and potential antimycobacterial strategies.

## INTRODUCTION

Tuberculosis (TB), caused by the Gram-positive bacillus Mycobacterium tuberculosis, remains a serious threat in our living environments due to the emergence of single-drug resistance and multiply drug-resistant strains (1). Treatment of drug-resistant M. tuberculosis strains is particularly challenging and requires the identification of novel targets and also the development of effective drugs (2). It was previously reported that M. tuberculosis can survive and obtain its basic needs in the acidic hypoxic environment of the hostile macrophage and that cholesterol of macrophage may serve an important function (3). During the infection, host cholesterol is suggested to promote the entry of mycobacteria into macrophages and to be important for its persistence in the lungs of chronically infected mammals (4). Host cholesterol is then metabolized through the initial degradation of the aliphatic side chain and the subsequent degradation of sterol rings A to D by a series of adenylating enzymes in TB strains (4). After that, metabolic pools of propionyl-coenzyme A (CoA) and acetyl-CoA increase in volume and are used as sources of carbon and of energy for the metabolic demands of TB strains and also as building blocks of multimethyl-branched mycolic acids operating through glyoxylate cycle, methylcitrate cycle, and methylmalonyl pathways, respectively (5). Therefore, metabolic enzymes in the cholesterol metabolism have become potential therapeutic targets (6). It seems that elucidation of the dynamic features of enzyme expression during mycobacterial cholesterol metabolism is essential.

Acyl-CoAs, such as acetyl-CoA and propionyl-CoA, are also donors of lysine acylation. Different types of lysine acylation are highly related to diverse biological functions, such as epigenetics, cellular metabolism, signal transduction, cell cycle, and aging, and are important in both prokaryotes and eukaryotes (7, 8). Owing to the mass spectrometry (MS)-based proteomic analyses performed in recent years, we and other groups have expanded knowledge of lysine acylomes in various bacteria, including Escherichia coli, Pseudomonas aeruginosa, Vibrio parahaemolyticus, Thermus thermophilus, Saccharopolyspora erythraea, and Mycobacterium tuberculosis (9–12). In those studies, lysine acylations were proven to modify virulence factors and to be involved in bacterial pathogenesis (13). Therefore, full understanding of the lysine acylation feature in pathogens is essential for the development of novel therapeutic agents against pathogenic bacterial infection.

Until now, some omic studies have explored the changes in the process of adaptation of mycobacteria to the cholesterol environment at the transcriptome and metabolome levels (14, 15). We and other groups also reported results of the proteomic characterization of lysine acetylation, propionylation, succinylation, and phosphorylation in different mycobacterial species (16–19), as well as the influence of lysine acylation on protein activities, DNA binding, and protein stability (20, 21). A recent study reported regulation of lysine acetylation in tuning the phosphotransfer ability of two response regulator proteins, TcrX and MtrA, in M. tuberculosis (22). Another study uncovered the dynamic changes in lysine acetylation and phosphorylation in the same perturbations in the pathogenic bacterium Mycoplasma pneumoniae (20). Therefore, comprehensive understanding of the cross talk between different lysine acylations or between lysine acylations and other posttranslational modifications (PTMs) may increase our current knowledge. However, the dynamic features of mycobacterial cholesterol metabolism at the protein expression level or the PTM level remain unknown. In this study, using nonpathogenic Mycobacterium smegmatis as a model, we mainly presented the global picture of protein expression, acetylation, propionylation, and phosphorylation changes for M. smegmatis growing in cholesterol compared with its growth in glucose. Our results revealed that almost all enzymes involved in mycobacterial cholesterol metabolism and several metabolic enzymes catalyzing acetyl-CoA/propionyl-CoA utilization showed higher expression in the presence of cholesterol. We also observed cholesterol-induced elevation of global levels of lysine acetylation and propionylation, with several adenylating enzymes involved in cholesterol metabolism shown to be acylated by the acyltransferase M. smegmatis Kat (*Ms*Kat). Furthermore, quantitative proteome and phosphorylome data showed that cholesterol changes also influenced levels of protein expression and phosphorylation of protein kinases, including PknA, PknB, and PknK. This comprehensive omic profiling functionally connected the specific characterization of cholesterol assimilation with the proteomic and PTM consequences in mycobacteria, which contributed to understanding of the relationship between pathogen infection and mycobacterial cholesterol degradation and provided new insights into potential antimycobacterial strategies.

## RESULTS

### Quantitative proteomic profile of cholesterol catabolism in M. smegmatis.

**FIG 1:**
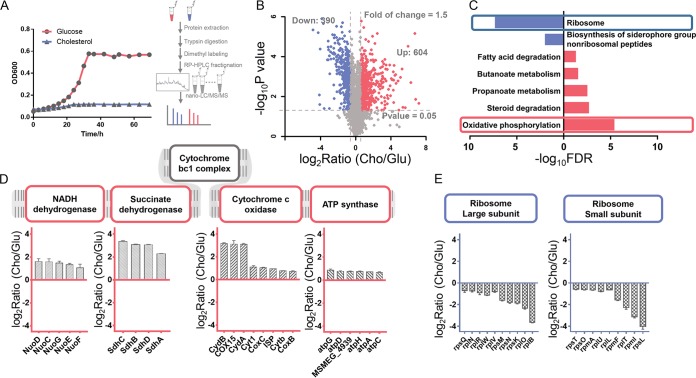
Quantitative proteomic analysis of the protein expression level in M. smegmatis in the presence of glucose or cholesterol. (A) Growth curve of M. smegmatis cultured under two sets of conditions and schematic illustration of the experimental process for quantitative proteomic analysis. RP-HPLC, reverse-phase high-performance liquid chromatography. (B) Volcano plots showing the changed proteins cultured in cholesterol (Cho) versus glucose (Glu). (C) KEGG enrichment analysis of the upregulated proteins and downregulated proteins. FDR, false-discovery rate. (D) Bar plot showing the upregulated proteins identified in the oxidative phosphorylation pathway. (E) Bar plot showing the downregulated proteins identified in ribosome.

To explore the changes in protein expression levels seen in the presence of glucose or cholesterol, we performed quantitative proteomics analyses using the labeling method employing the stable isotope dimethyl. After tryptic digestion, peptides from these two conditions were labeled “light” (CH_2_O and NaBH_3_CN) (for glucose) and “heavy” (CD_2_O and NaBH_3_CN) (for cholesterol) reagents, respectively (Fig. 1A). From the proteomic data, a total of 4,576 proteins were identified (see Data Set S1A and B in the supplemental material), among which 2,241 proteins were quantifiable (*P* value of <0.05 in three replicates) (Fig. 1B; see also Data Set S1B). The obviously changed proteins were defined according to the rules shown in Fig. S1A in the supplemental material and are summarized in Data Set S1B. The pathway enrichment analysis of the significantly changed protein indicated that proteins involved in the ribosome process and in biosynthesis of siderophore group nonribosomal peptides were downregulated and that proteins that participated in oxidative phosphorylation, propanoate metabolism, and steroid degradation were upregulated in the presence of cholesterol (Fig. 1C; see also Data Set S1C). We then analyzed the differently expressed proteins in the most highly enriched pathways, thus revealing that 23 proteins involved in oxidative phosphorylation were upregulated (Data Set S1C) and that 19 proteins involved in the ribosome process were downregulated (Data Set S1C). As shown in Fig. 1D, almost all enzymes in the four major complexes of oxidative phosphorylation were upregulated, which indicated that electron donor utilization in response to cholesterol was precisely controlled. When M. smegmatis switched to a nonreplicating state, the expression levels of several core ribosomal proteins responsible for protein synthesis decreased (Fig. 1E). These results were consistent with the phenotype that the growth rate in cholesterol was delayed compared with that in glucose (Fig. 1A).

10.1128/mSystems.00424-19.1 FIG S1 (A) Illustration of the statistics analysis used in the proteomic analysis of protein expression levels used in this study. (B) Illustration of the statistics analysis used in the PTMome analysis used in this study. Download FIG S1, TIF file, 0.7 MB. Copyright © 2020 Xu et al. 2020 Xu et al. This content is distributed under the terms of the Creative Commons Attribution 4.0 International license.

10.1128/mSystems.00424-19.8 DATA SET S1 (A) List of identified proteins in M. smegmatis (R1, R2, and R3). (B) List of proteins quantitatively analyzed in three replicates with *P* values of <0.05 and list of upregulated proteins and downregulated proteins. (C) KEGG enrichment analysis of upregulated or downregulated proteins. (D) Quantitative analyses of expression levels of protein derived from the proteomic data (from our study) and transcriptome data (from a previous study). Download Data Set S1, XLSX file, 5.5 MB. Copyright © 2020 Xu et al. 2020 Xu et al. This content is distributed under the terms of the Creative Commons Attribution 4.0 International license.

### Dynamic changes of cholesterol-related virulence factors in M. smegmatis.

Several important virulence factors whose inactivation failed to disturb the growth of mycobacteria but impaired mycobacterial pathogenicity were identified in mycobacteria (23). We then characterized the dynamic protein expression levels of these virulence factors by dividing them into four categories based on their function as follows: (i) cholesterol transport and degradation, (ii) cholesterol-derived carbon utilization, (iii) metal importers and exporters, and (iv) sigma factors.

**FIG 2:**
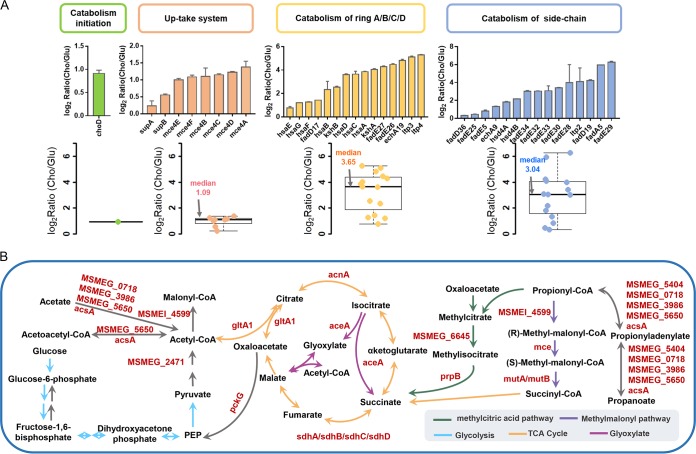
Dynamic changes in protein expression levels of enzymes in the transport and utilization pathways of mycobacterial cholesterol catabolism. (A) Bar plots and box plots showing protein expression levels of metabolic enzymes involved in the cholesterol intake and degradation pathway. (B) Presentation of upregulated proteins in glycolysis/glyconeogenesis, citrate cycle, propanoate metabolism, and glyoxylate and dicarboxylate metabolism. PEP, phosphoenolpyruvate.

Bioprocesses that included cholesterol uptake, catabolism, and utilization were necessary for mycobacterial virulence and pathogenesis during maintenance of the mycobacteria in the host macrophages (24). A transcriptional study of M. smegmatis growing in cholesterol or glycerol as the sole carbon source showed that expression of 454 genes was induced by cholesterol (25). In combination with our present data, the gene region (M. smegmatis MEG_5990 [MSMEG_5990] to MSMEG_6017) regulated by transcription factors KstR and KstR2 and involved in cholesterol catabolism as reported in a previous study (14) showed considerable correlation despite there being some experimental design differences (growth conditions and analysis platforms) between these two studies (Fig. S2A; see also Data Set S1D). This correlation coefficient was much higher than in results acquired in other transcriptomic/proteomic comparison analyses (Fig. S2B; see also Data Set S1D), which definitely indicated that cholesterol catabolic enzymes were upregulated in response to cholesterol. We present data representative of the enzymes in each cholesterol metabolism step, including the cholesterol up-taking system, sterol ring degradation, and side chain degradation, in Fig. 2A. In the cholesterol acquisition process, all the Mce4 proteins (Mce4A to Mce4F) were highly upregulated when cholesterol was used as the primary carbon source, with elevation levels higher than those seen with proteins in Mce1 and Mce2 families (Fig. S2C). This result confirmed that Mce4 was the major ABC-like transport system responsible for cholesterol import, which was consistent with previous reports (26). Similarly, metabolic enzymes involved in sterol rings A to D and aliphatic side chain degradation were also expressed at higher levels in the presence of cholesterol, including fatty acyl-CoA synthetases (FadD), acyl coenzyme A dehydrogenases (FadE), keto acyl-CoA thiolases (Ltp), and other ring-degrading enzymes (e.g., KsaAB and HsaA-G) (Fig. 2A).

10.1128/mSystems.00424-19.2 FIG S2 (A) Dot plot showing the correlation between proteome level and mRNA level of gene region MSMEG_5990 to MSMEG_6017. (B) Dot plot showing the correlation between proteome level and mRNA level of proteins identified in both platforms. (C) Bar and box plot of proteins identified in the MCE family. The whiskers of the box plot show the largest protein in 1.5 times the interquartile range. The bounds of the box show the upper and lower quartiles, and the line indicates the median. (D) Bar plot of proteins identified in the secretion system of M. smegmatis. Download FIG S2, TIF file, 0.8 MB. Copyright © 2020 Xu et al. 2020 Xu et al. This content is distributed under the terms of the Creative Commons Attribution 4.0 International license.

In the catabolism process involving steroid rings C and D, acetyl-CoA and propionyl-CoA were generated and then fed into the central tricarboxylic acid (TCA) cycle through the glyoxylate shunt and methylcitrate cycle (24). Propionyl-CoA could also be channeled into the detoxification pathway for the biosynthesis of virulence-associated lipids such as phthiocerol dimycoserate (PDIM) and sulfolipid-1 (SL-1) (24). The protein expression levels of metabolic enzymes catalyzing acetyl-CoA/propionyl-CoA assimilation were next analyzed. The β-glucoside-specific EII permease (MSMEG_2117), which catalyzed the uptake of extracellular glucose, was downregulated in the presence of cholesterol (Data Set S1B), whereas two important enzymes (glyceraldehyde-3-phosphate dehydrogenase [MSMEG_3084] and pyruvate kinase [MSMEG_3227]), which are involved in glucose intake and glycolysis, were expressed at lower levels in the presence of glucose (Data Set S1B). However, metabolic enzymes involved in the TCA cycle, glyoxylate shunt, methylcitrate cycle, and methylmalonyl pathway, including aconitate hydratase (MSMEG_3143), isocitrate lyase (MSMEG_0911), methylmalonyl-CoA epimerase (MSMEG_4921), methylmalonyl-CoA mutase (MSMEG_3158), methylisocitrate lyase (MSMEG_6646), and 2-methylcitrate dehydratase (MSMEG_6645), showed higher protein expression levels in the presence of cholesterol (Fig. 2B). This result strongly suggested that cholesterol-derived acetyl-CoA and propionyl-CoA were used as the major receptacles for storing energy, maintaining the survival of mycobacteria in host macrophage.

Mycobacterial secretion systems are vital for the adaptation and survival of the bacterium in its natural surroundings. Therefore, we explored the expression levels of the related proteins. The results showed that several proteins involved in the ESX-1 type secretion system, which were responsible for transferral of genetic material by conjugation, gained higher expression levels in the presence of cholesterol (Fig. S2D). The results indicated that the bacteria tended to use this system to export toxins/signals and to interact with host cells. Two mycobacterial sigma factors, sigma G (involved in the SOS response) and sigma H (involved in responses to oxidative, nitrosative, and heat stresses), were also upregulated in response to cholesterol (Data Set S1B), which was consistent with previous transcriptional assays (27). Meantime, no obvious changes were observed in the protein expression levels of mycobacterial polyketide synthases used for PDIM and SL-1 biosynthesis (Data Set S1B), preventing the bacteria from attack of host immunogenicity.

Together, these results provided a comprehensive cholesterol response assay at the proteomic level and revealed the upregulation of several cholesterol-induced metabolic enzymes involved in cholesterol absorption and degradation and absorption utilization.

### Elevated global acetylation and propionylation levels induced by cholesterol.

**FIG 3:**
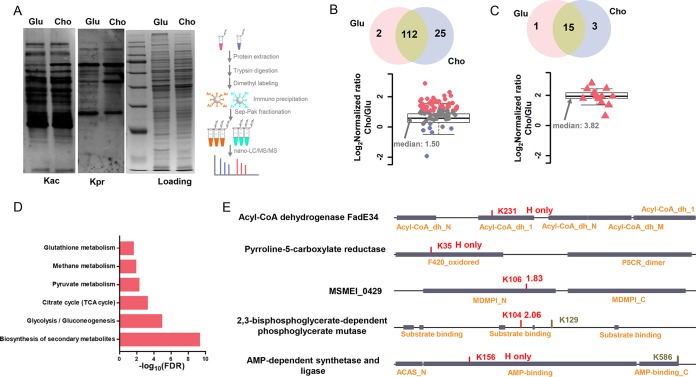
Quantitative analysis of lysine acetylome and propionylome in M. smegmatis cultured in the presence of glucose or cholesterol. (A) Western blot analysis of lysine acetylation or propionylation and schematic illustration of the experimental process for quantitative acetylome and propionylome analysis. (B) Venn diagram of identified lysine-acetylated substrates in the presence of glucose or cholesterol and box plot showing the quantitative acetylome data. (C) Venn diagram of identified lysine propionylated substrates in the presence of glucose or cholesterol and box plot showing the quantitative propionylome data. (D) KEGG enrichment analysis of upregulated acetylated substrates. (E) Annotation of acetylation substrates in M. smegmatis.

As mentioned above, metabolic alterations caused by cholesterol catabolism centered on acetyl-CoA and propionyl-CoA pools, which provided direct donors for lysine acetylation and propionylation. We thus investigated whether acetylation and propionylation levels might change on a set of modified substrates in response to cholesterol. Western blot analyses of levels of lysine acetylation (Kac) and propionylation (Kpr) of M. smegmatis growing in cholesterol or glucose were performed first. The results showed global elevation in the levels of both acetylation and propionylation (Fig. 3A). To further explore the acetylated and propionylated substrates, we conducted quantitative acylome analysis using the stable isotope dimethyl labeling method (Fig. 3A) and manually checked all spectra for quality control. A total of 139 Kac sites in 102 proteins and 19 Kpr sites in 18 proteins were identified (Data Set S2A and B and S2D and E). Seven lysine residues and 9 protein substrates were shared between lysine acetylation and propionylation, respectively (Fig. S3). We then performed qualitative and quantitative analyses of acylated lysine substrates. The results presented in Fig. 3B and C show that the median H/L (glucose condition/cholesterol condition) ratio was 1.5 for acetylome data and 3.82 for propionylome data (after protein level normalization). Our proteomic data were highly consistent with the data from the Western blot assay, which indicated that cholesterol induced both hyperacetylation and hyperpropionylation in M. smegmatis. The obviously changes in the acetylated and propionylated substrates were defined according to the rules listed in Fig. S1B and summarized in Data Set S2B and E. Pathway enrichment analysis of protein substrates with elevated acetylation level indicated obvious enrichment of glycolysis/gluconeogenesis, citrate cycle, pyruvate metabolism, methane metabolism, propanoate metabolism, and glutathione metabolism (Fig. 3D; see also Data Set S2C). Several key lysine residues in the functional domains of hyperacetylation proteins were also provided, including K231 in FadE34, K35 in pyrroline-5-carboxylate reductase, K106 in MSMEI_0429, K104 in 2,3-bisphosphoglycerate-dependent phosphoglycerate mutase, and K156 in AMP-dependent synthetase/ligase (Fig. 3E). This result suggested a possible role of regulation of acetylation in the protein substrates and the regulation function of cholesterol in the protein expression level as well as in the lysine acylation level.

10.1128/mSystems.00424-19.3 FIG S3 Venn diagrams of identified lysine acetylation and lysine propionylation in lysines and proteins. Left, acylated sites; right, acylated proteins. Download FIG S3, TIF file, 0.1 MB. Copyright © 2020 Xu et al. 2020 Xu et al. This content is distributed under the terms of the Creative Commons Attribution 4.0 International license.

10.1128/mSystems.00424-19.9 DATA SET S2 (A) List of acetylated substrates identified in M. smegmatis (R1, R2, and R3). (B) List of quantitative acetylated substrates in three replicates with *P* values of <0.05 and list of upregulated and downregulated acetylated substrates. (C) KEGG enrichment analysis of the upregulated acetylated proteins. (D) List of propionylated substrates identified in M. smegmatis (R1, R2, and R3). (E) List of quantitative propionylated substrates in three replicates with *P* values of <0.05 and list of upregulated and downregulated propionylated substrates. Download Data Set S2, XLSX file, 0.2 MB. Copyright © 2020 Xu et al. 2020 Xu et al. This content is distributed under the terms of the Creative Commons Attribution 4.0 International license.

### Mechanism and regulation of mycobacterial fatty acyl-CoA synthetase FadD35.

**FIG 4:**
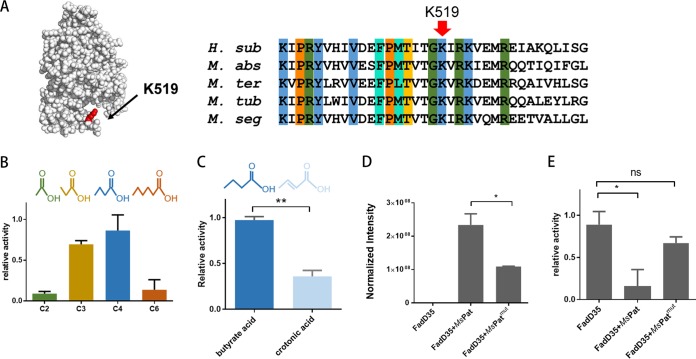
Mechanism and enzymatic regulation assays of mycobacterial fatty acyl-AMP ligase FadD35. (A) Visualization of the propionylated site in the constructed crystal structure of FadD35 and sequence alignment of orthologous proteins from Hoyosella subflava (*H. sub*), Mycobacteroides abscessus (*M. abs*), Mycolicibacter terrae (*M. ter*), and Mycobacterium tuberculosis (*M. tub*) and M. smegmatis (*M. seg*). (B) Relative enzymatic activity of FadD35 for acetic acid (C2), propionic acid (C3), n-butyric acid (C4), and n-hexylic acid (C6) as substrates. (C) Relative enzymatic activity of FadD35 for butyrate acid and crotonic acid as substrates (**, *P* value < 0.01, Student's *t* test). (D) Mass spectrum analysis showing the propionylation level of FadD35 with the catalysis of *Ms*Pat or *Ms*Pat^mut^ (*, *P* value < 0.05, Student's *t* test). (E) Relative enzymatic activity of FadD35 for n-butyric acid (C4) with the catalysis of *Ms*Pat or *Ms*Pat^mut^ (*, *P* value < 0.05; ns, *P* value > 0.05, Student's *t* test).

Mycobacteria encode more than 60 adenylating enzymes, catalyzing a series of cellular processes in lipid metabolism, glycolysis, cofactor biosynthesis, and special metabolites (e.g., mycobactins and mycothiols) (28). Among them, fatty acyl-CoA ligases showed broad substrate specificity and synthesized CoA esters from short to long-chain fatty acids. Due to their necessity in cholesterol catabolism and biosynthesis of mycobacterial lipids, fatty acyl-CoA ligases have become attractive targets for development of new anti-TB agents (6). The proteomic data revealed that FadD35, one of the mycobacterial fatty acyl-CoA ligases, showed significantly higher propionylation levels at the conserved K519 sites in the presence of cholesterol (Fig. 4A). To investigate the regulatory mechanism of propionylation on FadD35, we first explored the substrate specificity of FadD35 using fatty acids of different lengths from C2 to C6. The four substrates were showed measurable activity levels, and butyrate acid (C4) showed the highest catalytic priority (Fig. 4B). In addition, we compared the catalytic activity of FadD35 with that of saturated and unsaturated C4 fatty acids. Our results showed a preference for butyrate acid over unsaturated crotonic acid (Fig. 4C). FadD enzymes, including FadD4, FadD32, and FadD33, were previously reported to be the bona fide substrates of the cAMP-dependent acyltransferase *Ms*Kat in M. smegmatis (18, 29); thus, we speculated whether FadD35 could also be propionylated by *Ms*Kat with propionyl-CoA as the donor. Using a R95K/E234A *Ms*Kat (mutant *Ms*Kat) double mutant as a negative control (30), the results indicated that FadD35 was propionylated in the presence of *Ms*Kat whereas much lower propionylation levels were observed in the catalysis of the mutant *Ms*Kat (Fig. 4D). Similar results were observed when peptides were used as substrates (Fig. S4). We also evaluated the AMP ligase activity of FadD35 with or without wild-type *Ms*Kat or mutant *Ms*Kat. The result clearly showed that wild-type *Ms*Kat impaired the enzymatic activity of FadD35 whereas mutant *Ms*Kat had no obvious effect (Fig. 4E). Together, our results revealed the details of the enzymatic mechanism of FadD35 and further proved that adenylating of FadD35 was regulated by the cAMP-dependent *Ms*Kat protein.

10.1128/mSystems.00424-19.4 FIG S4 Mass spectrum analysis showing the propionylation level of FadD35 with the catalysis of *Ms*Pat or *Ms*Pat^mut^. Left, 2+ substrate peptides; right, 3+ substrate peptides. Download FIG S4, TIF file, 0.3 MB. Copyright © 2020 Xu et al. 2020 Xu et al. This content is distributed under the terms of the Creative Commons Attribution 4.0 International license.

### Lysine acylation inhibits the catalytic activity of other adenylating enzymes.

**FIG 5:**
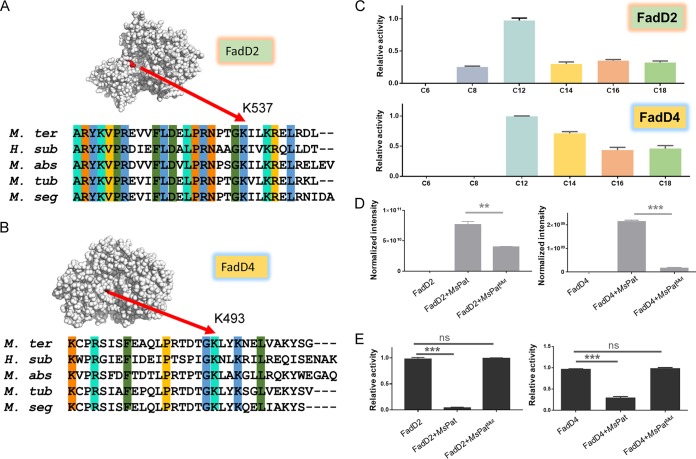
Substrate characterization and regulation features of other adenylating enzymes. (A) Visualization of the propionylated site in the constructed crystal structure of FadD2 and sequence alignment of orthologous proteins from Hoyosella subflava (*H. sub*), Mycobacteroides abscessus (*M. abs*), Mycolicibacter terrae (*M. ter*), and Mycobacterium tuberculosis (*M. tub*) and M. smegmatis (*M. seg*). (B) Visualization of the propionylated site in the constructed crystal structure of FadD4 and sequence alignment of orthologous proteins from Hoyosella subflava, Mycobacteroides abscessus, Mycolicibacter terrae, Mycobacterium tuberculosis, and M. smegmatis. (C) Relative enzymatic activity of FadD2 or FadD4 for fatty acids of different lengths. (D) Mass spectrum analysis showing the propionylation level of FadD2 (left) or FadD4 (right) with the catalysis of *Ms*Pat or *Ms*Pat^mut^ (**, *P* value < 0.01; ***, *P* value < 0.001, Student's *t* test). (E) Relative enzymatic activity of FadD2 (left) or FadD4 (right) for lauric acid (C12) with the catalysis of *Ms*Pat or *Ms*Pat^mut^ (***, *P* value < 0.001; ns, *P* value > 0.05, Student's *t* test).

According to the proteomics data, other FadD enzymes, including FadD2 and FadD4, were also hyperpropionylated in response to cholesterol. The modeling and sequence alignment analyses indicated that K537 of FadD2 and K493 of FadD4 were the conserved enzymatic activity sites (Fig. 5A and B). Both FadD2 and FadD4 showed a higher preference for C12 fatty acids (Fig. 5C). Similarly to FadD35, mutant *Ms*Kat failed to propionylate the active site of both FadD2 and FadD4 in comparison with the wild-type *Ms*Kat (Fig. 5D) and had no effect on the activity of both FadD2 and FadD4 in response to the catalysis of mutant *Ms*Kat (Fig. 5E), and addition of acetyl-CoA decreased the relative propionylation level of K537 in FadD2 and of K493 in FadD4 (Fig. S5A and B). The phenomenon of competition experiment was similar to the results acquired in other *Ms*Kat substrates, such as USP protein, acetyl-CoA synthetase, and propionyl-CoA synthetase (18). Furthermore, another suspected adenylating enzyme, encoded by gene *MSMEI_4013*, was found to bear the conserved lysine residues of FadD enzyme family (Fig. S5C) and to share the feature of cholesterol-induced hyperpropionylation. The description in the KEGG database showed that it might possibly represent a cyclohexanecarboxylate-CoA ligase which catalyzed the thioester reaction with cyclized cyclohexanecarboxylate as the substrate. Our enzymatic assays further proved that it was also the bona fide substrate of *Ms*Kat (Fig. S5D) and that the competition of acetyl-CoA with propionyl-CoA affected the propionylation level of K533 (Fig. S5E). A number of studies showed that cholesterol serves as the primary energy source during nutrient restriction in the host macrophage (31). Propionyl-CoA degradation resulting from the presence of cholesterol was toxic to the strain, and the major detoxication mechanism was mediated through the methylcitrate cycle in mycobacteria (32). Our quantitative propionylome results strongly suggested that during the cholesterol catabolism, cellular propionyl-CoA could be utilized by acyltransferase *Ms*Kat for propionylation, regulating the cellular propionyl-CoA pools and thereby alleviating its toxicity. Therefore, targeting acyltransferase *Ms*Kat in addition to the FadD enzymes themselves might represent a potential design for an anti-TB strategy.

10.1128/mSystems.00424-19.5 FIG S5 (A, B, and E) MS analysis showing the relative acetylation/propionylation levels of key lysine residues. (Left) Relative acetylation levels of key lysine residues in the presence of acetyl-CoA and/or propionyl-CoA. (Right) Relative propionylation levels of key lysine residues in the presence of propionyl-CoA and/or acetyl-CoA. (A) K537 of FadD2. (B) K493 of FadD4. (E) K533 of MSMEI_4013. (C) Visualization of the propionylated site in the constructed crystal structure of MSMEI_4013 and sequence alignment of FadD2, FadD4, FadD35, PrpE, and MSMEI_4013. (D) Mass spectrum analysis showing the propionylation level of MSMEI_4013 with or without propionyl-CoA, cAMP, and *Ms*Pat. Peptides with different charges (2^+^, 3^+^, or 4^+^) or modifications (oxidation of methionine or not) are presented. Download FIG S5, TIF file, 2.4 MB. Copyright © 2020 Xu et al. 2020 Xu et al. This content is distributed under the terms of the Creative Commons Attribution 4.0 International license.

### Characterization of cholesterol-based dynamic changes of other PTMs in M. smegmatis.

**FIG 6:**
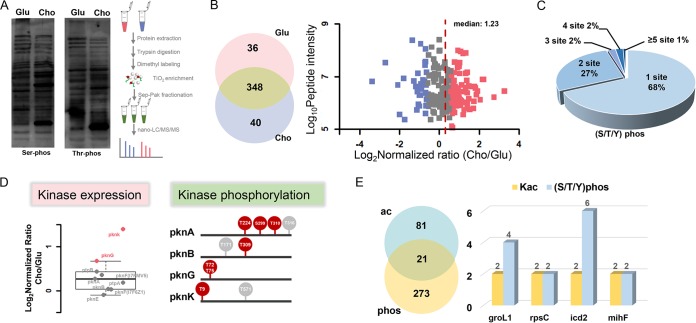
Quantitative analysis of S/T/Y phosphorylome in M. smegmatis cultured in the presence of glucose or cholesterol. (A) Western blot analysis of serine phosphorylation (Ser-phos) or threonine phosphorylation (Thr-phos) and schematic illustration of the experimental process for quantitative phosphorylome analysis, and the loading control was the same as that shown in Fig. 3A. (B) Venn diagram of identified serine/threonine/tyrosine (S/T/Y) phosphorylation in M. smegmatis growing in cholesterol or glucose and dot plot showing quantification of phosphorylated protein. (C) Presentation of proteins with different numbers of phosphorylated sites. (D) Protein expression levels of kinases and their phosphorylation levels. (E) Venn diagram of identified acetylation and phosphorylation proteins and bar plot showing identified lysine acetylation and S/T/Y phosphorylation sites in GroL1, RpsC, Icd2, and MihF.

Other forms of lysine modification have been reported to regulate mycobacterial features. Lysine succinylation, which was identified by us and other groups (33), broadly regulated diverse metabolic pathways in mycobacteria, such as glycolysis, fatty acid oxidation, TCA cycle, and mycolic acid biosynthesis (17). Lysine pupylation, catalyzed by a prokaryotic ubiquitin-like protein, was the direct target of degradation by the bacterial proteasome (34). A Pup-proteasome system was previously shown to be essential for mycobacterial virulence and persistence in the host macrophage (35). In addition to lysine modifications, mycobacteria encoded 11 Ser/Thr/Tyr protein kinases and 2 Ser/Thr/Tyr phosphatases (19, 36). The Ser/Thr/Tyr phosphorylation represented essential functions of coordinate regulation in hostile environments during mycobacterial infection (37). We thus performed Western blot analysis and found obvious difference in serine and threonine phosphorylation levels between glucose and cholesterol conditions (Fig. 6A). Using quantitative phosphorylome assays performed with the TiO_2_-based enrichment method, 72 upregulated phosphorylated peptides (58 proteins) and 53 downregulated phosphorylated peptides (49 proteins) were observed in all 368 substrates (294 proteins) (Fig. 6B; see also Data Set S3A and B). In addition, over 30% of the phosphorylated substrates bore two or more phosphorylated sites (Fig. 6C). The results indicated that changes in carbon metabolism (from glucose to cholesterol) resulted in simultaneous perturbations of cellular proteome, lysine acylation, and Ser/Thr/Tyr phosphorylome. Our phosphorylome data revealed that T224, S229, and T310 in PknA; T309 in PknB; and T9 in PknK showed much higher phosphorylation levels in the presence of cholesterol (Fig. 6D). Meantime, the proteomic data revealed that most of the mycobacterial kinases identified showed relatively higher protein expression levels (Fig. 6D). On the basis of these data, kinases regulated at both the proteomic and phosphorylation levels might represent additional regulatory factors that are essential for sustaining mycobacterial survival in host macrophages. In addition to kinases, other substrate proteins exhibited different changes in their phosphorylation levels in response to carbon shift. For example, T212 was located in the NAD binding domain of AhcY, and its phosphorylation level decreased during growth on cholesterol. Similar dynamic phosphorylated patterns were observed for T17 in GarA, T140 and S142 in PmmB, T2, T15, and T37 in Rho, etc. (Data Set S3B). In contrast, several serine/threonine/tyrosine sites were identified with elevated phosphorylation levels in the presence of cholesterol, including T7 in MihF, T869 and T979 in MmpL3, S275 in RpsC, etc. (Data Set S3B). We identified 21 proteins that could be both acetylated and phosphorylated (Fig. 6E). Among them, GroL1, RpsC, Icd2, and MihF contained no less than two acetylated sites and phosphorylated sites (Fig. 6E). The model structure also showed the space distance between the acetylated lysine and phosphorylated serine/threonine in *groL1* and *ppgK*, indicating their potential interactions (Fig. S6A and B).

10.1128/mSystems.00424-19.6 FIG S6 (A) Constructed crystal structure of A0QQU5 by homology modeling (PDB: 5DA8, 60% identity). The modified amino acids are shown using a stick model. (B) Constructed crystal structure of I7G9D5 by homology modeling (PDB: 1WOQ, 49.39% identity). The modified amino acids are shown using a stick model. (C) Summary of this study. Quantitative proteome, acetylome, propionylome, and phosphorylome analyses of M. smegmatis growth in cholesterol in comparison with growth in glucose were performed. Download FIG S6, TIF file, 2.7 MB. Copyright © 2020 Xu et al. 2020 Xu et al. This content is distributed under the terms of the Creative Commons Attribution 4.0 International license.

10.1128/mSystems.00424-19.10 DATA SET S3 (A) List of phosphorylated substrates identified in M. smegmatis (R1, R2, and R3). (B) List of quantitative phosphorylated substrates in three replicates with *P* values of <0.05 and list of upregulated and downregulated phosphorylated substrates. (C) List of pupylated substrates in three replicates with *P* values of <0.05. (D) List of succinylated substrates in three replicates with *P* values of <0.05. Download Data Set S3, XLSX file, 0.5 MB. Copyright © 2020 Xu et al. 2020 Xu et al. This content is distributed under the terms of the Creative Commons Attribution 4.0 International license.

Furthermore, we searched the potential substrates and estimated the levels of their dynamic changes in lysine succinylation and pupylation using the quantitative protein profiling data. Seven succinylated sites and 11 pupylated sites were identified with high-quality spectra (Data Set S3C and D). Due to the much lower intensity of the modified peptides, no valuable quantitative information could be acquired from our proteomic data. The exploration of lysine succinylation and pupylation by antibody enrichment is planned to be conducted in our further study. Our extensive analysis proved that perturbations in the carbon source (shift from glucose to cholesterol) resulted in modulation of kinase abundance and substrate S/T/Y phosphorylation patterns, revealing potential interplay of protein acylation with other posttranscriptional regulatory mechanisms.

## DISCUSSION

In recent years, efforts aimed at the discovery of novel anti-TB drugs encountered several obstacles, and the chemical agents investigated have limitations, including low efficiency and oral bioavailability and potential side effects (38). Cholesterol of host membrane was found to represent more than a simple carbon source and was proven to be assimilated by mycobacteria to provide energy and to be converted to pathogenic bacterial products. Therefore, selective inhibitors were developed to target enzymes within the mycobacterial cholesterol pathway (39), and these chemical interventions were shown to induce carbon limitation, metabolic intoxication, and perturbation of central metabolism in mycobacteria (40). However, the complex cellular networks influenced by cholesterol were found to be involved at the metabolic, transcriptional, translational, and posttranslational levels and to be highly related to different environmental factors, including metabolic enzymes and their dynamic PTMs. Recent studies using metabolomic and transcriptomic profiling preliminarily connected features of the intracellular environment with gene expression and metabolic consequences in mycobacteria (14, 15, 25).

Culture of mycobacteria in cholesterol has been used to mimic the state of dormancy to explore the gene expression and regulatory mechanisms (41). Cholesterol controlled at a concentration of 0.01% has been widely used to culture the mycobacterium *in vitro*, while 10 mM glucose was used as a control (15, 26, 42, 43). Our results showed that exposure to cholesterol induced proteome change, including elevated protein levels in such biological functions as those involving steroid degradation (Fig. 2A), the mammalian cell entry (MCE) operon (see Fig. S2C in the supplemental material), and RD1 (Fig. S2D), which were previously reported to be important virulence factors of mycobacteria (23). Meantime, other important virulence factors, including Icl1, Acr1, and Mycp1, were also highly expressed in the presence of cholesterol, reflecting the higher pathogenicity of mycobacteria grown in cholesterol.

M. smegmatis is a fast-growing and nonpathogenic species, and several pathways are conserved between M. smegmatis and M. tuberculosis (44). In the cholesterol metabolism pathway (KEGG pathway accession no. mtu00984) of M. tuberculosis, 12 of the total of 13 enzymes have homology in M. smegmatis. The results thus indicate that the findings regarding cholesterol metabolism within M. smegmatis may be extended to M. tuberculosis. We conducted quantitative proteome, acetylome, propionylome, and phosphorylome analyses of M. smegmatis growing in cholesterol in comparison with growth in glucose (Fig. S6C). Our results showed that metabolic enzymes involved in mycobacterial cholesterol catabolism were expressed at higher levels in the presence of cholesterol. Also, we found that cholesterol induced elevation of lysine acetylation and propionylation levels and that several adenylating enzymes (e.g., FadD35) involved in cholesterol metabolism were regulated by *Ms*Kat-dependent lysine propionylation.

Our results showed that both protein expression levels and lysine acylation levels of metabolic enzymes in cholesterol-related pathways changed in an obvious manner during the metabolic shift from glucose to cholesterol. Previous studies proved that accumulation of propionyl-CoA results in metabolic disorders and even cytotoxicity in microorganisms (45), and we found that mycobacteria could adopt three major strategies to avoid the overflow of cellular propionyl-CoA. Assimilation of propionyl-CoA for energy supply and for building blocks of methyl branched lipid or mycolic acids could be the major two strategies, resulting in decreased concentrations of formed propionyl-CoA. And these two strategies were regulated at the protein expression level, with several related enzymes involved in the methylcitrate cycle and methylmalonyl pathway upregulated as shown in Fig. 2B. The third strategy was regulated at the PTM level, in which some essential adenylating enzymes vital for propionyl-CoA generation from cholesterol catabolism such as FadD2, FadD4, and FadD35 were inhibited by *Ms*Kat-mediated lysine propionylation. This strategy could block the further biosynthesis of propionyl-CoA. It seemed conceivable that acyl-CoA (e.g., propionyl-CoA) homeostasis might reflect the intricate interplay between beneficial and adverse effects of this cholesterol-derived metabolite. Therefore, potential chemical interference resulting in an imbalance of cellular acyl-CoA pools could represent a potential anti-TB drug design.

Reverse lysine acetylation and Ser/Thr/Tyr phosphorylation have broad regulatory functions, and lysine acetyltransferases and protein kinases were previously reported to play pivotal roles in the survival and pathogenicity of bacteria (46–49). Our results revealed the perturbation of carbon metabolism (from glucose to cholesterol) on multiple PTMs, and further model structure analysis and MS experimentation suggested the potential for cross talk between these different PTMs (Fig. S5A, B, and E and S6A and B), providing additional investigation details enhancing our knowledge of mycobacteria.

Increased cAMP concentrations were shown to block early stages in the cholesterol degradation, which disturbed mycobacterial replication in macrophages (39). Our results provided different insights revealing that the cAMP-dependent acyltransferase *Ms*Kat inhibited the active lysine by elevating the acylation levels of several different adenylating enzymes, which might slow the rate of utilization of cholesterol. That activity might represent a self-protection mechanism for mycobacteria and protect mycobacteria from attacks by the host immune system. Therefore, manipulation of cellular cAMP concentrations requires further consideration for designing new anti-TB strategy.

Moreover, our results also proved that systematic perturbations of carbon source also disturbed other layers of regulation, such as acylation and S/T/Y phosphorylation and their regulatory enzymes. Higher protein expression levels and phosphorylation levels of several mycobacterial kinases were observed in the presence of cholesterol. A previous study also reported that mycobacterial kinases interacted with proteins engaged in diverse functions such as cell wall integrity (50). It was suggested that utilization of kinase inhibitors might be another possible strategy for the potential treatment of chronic mycobacterial infection.

Our study gave new insight into the penetration of signals through the metabolic shift from glucose to cholesterol, providing an understanding of pathogen regulation and the foundation for efforts aimed at the discovery of new therapeutic agents in mycobacterial cholesterol catabolism.

## MATERIALS AND METHODS

### Cell culture and protein extraction.

Mycobacterium smegmatis MC2 155 was cultured in 5 ml Luria-Bertani (LB) media supplemented with 0.5% Tween 80 at 37°C. Cells were then washed with phosphate-buffered saline (PBS) and transferred to Middlebrook 7H9 liquid medium supplemented with either 10 mM glucose or 0.01% cholesterol. Cell growth levels were monitored by measuring optical density at 600 nm (OD_600_). Cells were collected at the middle stationary phase and then washed with PBS. Next, cells were lysed in lysis buffer (8 M urea supplemented with protease inhibitor cocktail [Calbiochem, Darmstadt, Germany] and 30 mM nicotinamide) on ice for 30 min and then sonicated for 5 min. Samples were centrifuged at 21,000 × *g* for 10 min, and the supernatants were used for protein concentration measurements and further analysis.

### Western blot analysis.

Protein (10 μg) was isolated by sodium dodecyl sulfate polyacrylamide gel electrophoresis (SDS-PAGE) and then transferred to a nitrocellulose filter membrane and maintained at 100 V for 90 min. Subsequently, the membrane was blocked by the use of B-PBST buffer (5% bovine serum albumin [BSA]–PBST buffer [PBS buffer with 0.1% Tween 20]) for 60 min. The membranes were then incubated with pan-antiacetyllysine antibody (PTM Biolabs, Hangzhou, China), pan-antipropionyllysine antibody (PTM Biolabs, Hangzhou, China), pan-antiphosphoserine antibody (ImmuneChem, Burnaby, Canada), or pan-antiphosphothreonine antibody (ImmuneChem, Burnaby, Canada) at 4°C overnight. The membranes were washed with PBST three times and then incubated with horseradish peroxidase (HRP)-conjugated anti-rabbit IgG (1:5,000)–PBST at room temperature for 1 h. Next, the membranes were washed with PBST three times and then treated with chemiluminescent HRP substrate (Millipore, Temecula, CA). After that, the samples were used for signal detection.

### In-solution trypsin digestion.

Protein extraction was reduced by the use of 5 mM dithiothreitol (DTT) at 56°C for 30 min. Subsequently, the sample was alkylated by the use of 15 mM iodoacetamide in darkness for 30 min and the process was terminated by the use of 30 mM cysteine for 30 min at 25°C. The sample was then incubated with trypsin (1:50 [wt/wt]) for 16 h at 37°C. A second digestion was performed with trypsin (1:100 [wt/wt]) for 4 h. The generated peptides were desalted and then dried by the use of a SpeedVac and used for dimethyl labeling.

### Stable isotope dimethyl labeling and HPLC fractionation.

Peptides were dissolved in 600 μl 100 mM triethylammonium bicarbonate buffer. Next, 30 μl 20% CH_2_O or CD_2_O with 30 μl 3 M NaBH_3_CN was added to sample to be “L” labeled (glucose condition) or “H” labeled (cholesterol condition), respectively. The reactions were performed for 60 min at room temperature. Ammonia (20%) was used as quencher. Finally, the samples were acidified by the use of trifluoroacetic acid (TFA), desalted through SepPak C_18_ cartridges, and dried using a SpeedVac. For protein profiling, 150-μg volumes of peptides were separated by the use of high-pH reverse-phase high-performance liquid chromatography (HPLC) performed with a Waters XBridge Prep C_18_ column (Waters Corp., Milford, MA) (19 by 150 cm) at a flow rate of 0.7 ml·min^−1^. Samples were collected and combined into 10 fractions. Finally, all samples were dried using a SpeedVac.

### Affinity enrichment of acetylated or propionylated peptides.

Peptides were dissolved in NETN buffer (100 mM NaCl, 17 mM EDTA, 50 mM Tris-HCl, 0.5% NP-40, pH 8.0). The mixtures were incubated with PBS prewashed acetylated/propionylated antibody beads at 4°C overnight with gentle shaking. After the flowthrough was removed, the beads were washed with NETN buffer four times, ETN buffer (100 mM NaCl, 17 mM EDTA, 50 mM Tris-HCl, pH 8.0) once, and H_2_O twice. The acetylated/propionylated peptides were eluted by the use of water with 0.2% TFA. After that, peptides were desalted in a C_18_ ZipTip column and then separated by the use of a gradient elution buffer and combined into three fractions. Finally, samples were dried using a SpeedVac.

### Enrichment of phosphorylated peptides.

Peptides were dissolved in 700 μl binding buffer (70% acetonitrile [ACN], 5% TFA, 8.3 mM lactic acid), and then incubated with TiO_2_ beads (GL Sciences Inc., Japan) for 40 min. After the enrichment process, the beads were washed in binding buffer 5 times, in washing buffer I (30% ACN, 0.5% TFA) 1 time, and in washing buffer II (30% ACN, 0.5% TFA) 2 times. After that, phosphorylated peptides were eluted and separated by a gradient (2%, 5%, 8%, 10%, and 40%) of ACN in 15% ammonium hydroxide and combined into 3 fractions. Finally, samples were dried using a SpeedVac.

### Nano-liquid chromatography–tandem mass spectrometry (nano-LC-MS/MS) analysis.

Samples were dissolved in solvent A (0.1% formic acid–2% acetonitrile–water) and injected into a manually packed reverse-phase C_18_ column (Dikma Technologies Inc., Lake Forest, CA) (10-cm length by 75-μm inner diameter; C_18_ resin with 3-μm particle size; 90-Å pore diameter) which was connected to a nano-HPLC system (Thermo Fisher Scientific, Waltham, MA). The samples were analyzed at the flow rate of 300 nl/min. For protein profiling, the gradient was performed for 60 min, including the use of 5% to 13% solvent B (0.1% formic acid–10% water–acetonitrile) for 20 min, 13% to 26% solvent B for 31 min, 26% to 45% solvent B for 5 min, 45% to 80% solvent B for 1 min, and 80% solvent B for 3 min. For acetylome, propionylome, and phosphoproteome, the gradient was 90 min, with 2% to 8% solvent B for 10 min, 8% to 15% solvent B for 10 min, 15% to 35% solvent B for 54 min, 35% to 45% solvent B for 10 min, 45% to 80% solvent B for 3 min, and 80% solvent B for 3 min. An Orbitrap Fusion mass spectrometer (Thermo Fisher Scientific) was used with a nanospray ion source in positive mode. The mass spectrometry (MS) parameters were as follows: the mass resolution was 120 K at a mass-to-charge ratio (*m*/*z*) of 200 for both MS1 and MS2. For MS1, an *m*/*z* range of 350 to 1,300 was scanned, with either a single charge or more than six charges discarded. The automatic gain control (AGC) targets were set at 5E5. For MS2, the ions with intensity levels higher than 5,000 were isolated and sequentially fragmented by the use of higher collision dissociation (HCD) with a normalized collision energy level of 32%. The dynamic exclusion duration was set at 30 s. Three technical replicates were performed for both profiling data and PTM data.

### Mass spectrometry data analysis.

Raw data were analyzed using MaxQuant (v 1.5.1.2). Data for M. smegmatis MC2 were downloaded from the UniProt proteomic database (strain ATCC 700084/MC2 155; release date, February 2017). The database combined database identifier (ID) UP000000757 and ID UP000006158. For protein profiling data, the maximum missed cleavage value was set to 2 and the maximum number of modifications per peptide was set to 3. Trypsin/P was set as the digestion enzyme. Carbamidomethy (C) was set as the fixed modification, and acetyl at the protein N terminus, oxidation (M), succinylation (K), and pupylation (K) were added as variable modifications. (We also explored potential protein succinylation and pupylation by using the profiling data.) False-discovery-rate thresholds for proteins, peptides, and modification sites were specified at a maximum value of 0.01. For acetylome, propionylome, or phosphoproteome data, acetyl (K), propionyl (K), or phosphor (STY) was added for variable modifications. For PTMs identified in our studies, acetylated, propionylated, and phosphorylated sites with localization probability values lower than 0.75 and peptides from reverse or potential contaminant were removed. The PTM ratios were normalized by the use of ratios corresponding to their protein levels and used for further analysis (51–54). Statistical analyses of PTM data were performed by using the two-tailed *t* test for the three replicates. All the txt folders which contained the detailed parameters for profiling data and PTM data were uploaded in the iProX database.

### Cloning, mutagenesis, and expression of substrate proteins in Escherichia coli.

All genes used in this study (MSMEG_5649, MSMEG_0599, and MSMEG_4013) were amplified from the genomic of M. smegmatis by PCR. The PCR products were then cloned into pET-28a (+) and confirmed by DNA sequencing. For double-site mutagenesis of *Ms*Pat (E234A and R95K), the two site-directed mutations were sequentially finished by homologous recombination. Finally, all plasmids were transformed into E. coli BL21(DE3). The strains that contained plasmid for MSMEG_0257 had been acquired in our previous study (18). The primers used in our study are indicated in Fig. S7 in the supplemental material.

10.1128/mSystems.00424-19.7 FIG S7 List of primers used in this study. Download FIG S7, TIF file, 0.2 MB. Copyright © 2020 Xu et al. 2020 Xu et al. This content is distributed under the terms of the Creative Commons Attribution 4.0 International license.

A single clone was cultured in 5 ml LB medium, and the medium were transferred to 50 ml LB medium supplemented with 0.1% kanamycin at 37°C. When the OD_600_ reached 0.6, cells were induced with 0.2 mM isopropyl-β-d-thiogalactopyranoside at 20°C overnight. The cells were then collected by centrifugation and washed with PBS. The cells were resuspended in PBS and sonicated for 10 min on ice. The mixture was centrifuged at 21,000 rpm for 20 min. The supernatant was loaded into a nickel-nitrilotriacetic acid (Ni-NTA) column (Merck). After washes were performed by the use of washing buffer (20 mM imidazole–50 mM NaH_2_PO_4_–300 mM NaCl, pH 8.0), the proteins were eluted by using the eluting buffer (250 mM imidazole–50 mM NaH_2_PO_4_–300 mM NaCl, pH 8.0). The purified proteins were dialyzed in PBS buffer and then concentrated through an Amicon Ultra-4-10K-molecular-weight-cutoff centrifugal device (Millipore). Protein concentration was measured using the bicinchoninic acid (BCA) method with PBS as a control.

### *In vitro* protein propionylation assays.

The substrates (4 μg) (MSMEG_5649, MSMEG_0599, or MSMEG_4013) were mixed with 30 μM propionyl-CoA and 1 mM cAMP in a reaction buffer (25 mM Tris-HCl [pH 7.5], 10 mM NaCl, 5 mM EDTA) at 37°C. The reaction was started by adding *Ms*Kat or its double mutant (E234A and R95K) and performed for 10 min. The reactions were terminated by the use of 2× loading buffer (100 mM Tris-HCl [pH 6.8], 4% [wt/vol] SDS, 20% [vol/vol] glycerol, 200 mM β-mercaptoethanol, 0.2% [wt/vol] bromophenol blue), and the mixtures were boiled for 1 min. Samples were then analyzed by SDS-PAGE.

### In-gel trypsin digestion and LC-MS/MS analysis.

Protein bands were cut from the gel and destained with 50% ethanol. After that, the band samples were washed with water and cut into small particles. The gel particles were then dehydrated by the use of ACN and dried using a SpeedVac. Next, gel particles were reduced with 10 mM DTT at 56°C and then alkylated in 55 mM iodoacetamide in darkness at room temperature (RT). The gel particles were then washed with 25 mM ammonium bicarbonate–50% ACN and dried using a SpeedVac. Subsequently, trypsin was added to the particles and the samples were incubated at 37°C for 16 h. Tryptic peptides were extracted by the use of a gradient of extraction buffer (50%, 75%, and 100% ACN with 0.1% TFA). The peptides were then pooled and dried using a SpeedVac.

The samples were dissolved in solvent A and then analyzed by the use of a Q Exactive mass spectrometer (Thermo Fisher Scientific) with a 60-min gradient (8% solvent B for 1 min, 8% to 43% solvent B for 45 min, 45% to 80% solvent B for 5 min, 80% solvent B for 10 min) at a flow rate of 300 nl/min. The MS parameters were as follows: for MS1, ions with *m*/*z* 350 to 1,300 and charge values of 2^+^ to 4^+^ were scanned. AGC was set at 5E5. The resolution was 70 K at *m*/*z* 200. For MS2, the top 16 most abundant ions with intensity values above 17,000 were fragmented by the use of higher collision dissociation (HCD) with normalized collision energy of 28%. The isolation window was 1.5 *m*/*z*, and the dynamic exclusion duration was 40 s.

The raw data were transformed to mgf files by the use of Thermo Proteome Discoverer (Thermo Fisher Scientific, v1.4.0.288) and then analyzed by Mascot (Matrix Science, v2.3). The parameters used for mascot search were as follows: The enzyme was trypsin/P. Maximum missed cleavage was set at a value of 2. Carbamidomethy (C) was set as a fixed modification, and acetyl (protein N terminus), oxidation (M), and propionyl (K) was set as variable modifications. Mass error settings of 10 ppm for MS and 0.02 Da for MS/MS were used. The M. smegmatis MC2 database was used as mentioned above in the searching process (MaxQuant software). Peptides with an ion score higher than 20 were manually checked, and propionylated peptides were analyzed. For quantification of propionylated peptides, *m*/*z* ratios of target peptides were used to determine peaks corresponding to each extracted precursor ion, and the area under the curve (AUC) normalized by the AUC of an unmodified peptide from the same protein was calculated. Two technical replicates were performed.

### Measurement of enzymatic activity of fatty acyl-CoA synthetase.

Fatty acyl-CoA synthetase activity was monitored by incubation using enzyme with 300 μM fatty acids in the reaction buffer (150 mM Tris-HCl [pH 7.2], 10 mM MgCl_2_, 2 mM EDTA, 0.1% Triton X-100, 5 mM ATP) at 37°C with PBS as the control. The reaction was initiated by addition of 0.5 mM CoA. Then, equal volumes of reaction buffer were picked and transferred into detection buffer {0.4 mM DTNB [5,5′-dithiobis(2-nitrobenzoic acid)] and 0.1 M Na_2_HPO_4_, pH 8.0}. The absorbance at *A*_412_ was then measured and used for calculating enzymatic activity. Two replicates were performed for each experiment.

### Modeling analysis of fatty acyl-CoA synthetase.

Protein crystal structures were adapted by using homolog modeling with the Swiss Model server and were visualized by the use of PyMol (v 1.8.6.1). Specifically, crystal structures of FadD35 (MSMEG_5649) were adapted from 5WM2 (Protein Data Bank [PDB] identifier), crystal structures of FadD2 (MSMEG_0599) were adapted from 3R44 (PDB), and crystal structures of FadD4 (MSMEG_0257) were adapted from 4GXR (PDB).

### Data availability.

The mass spectrometry proteomics data and spectra for modified peptides have been deposited to the ProteomeXchange Consortium (http://proteomecentral.proteomexchange.org) via the iProX partner repository with the data set identifier PXD014366. The mass spectrometry proteomics data have also been deposited at iProX with the data set identifier IPX0001659000.
