# Supplementary figures and images for: Dynamic Characterization of Protein and Posttranslational Modification Levels in Mycobacterial Cholesterol Catabolism
Source: mSystems. 2020 Jan 7;5(1):e00424-19. doi: 10.1128/mSystems.00424-19 (PMC6946793; doi:10.1128/mSystems.00424-19)

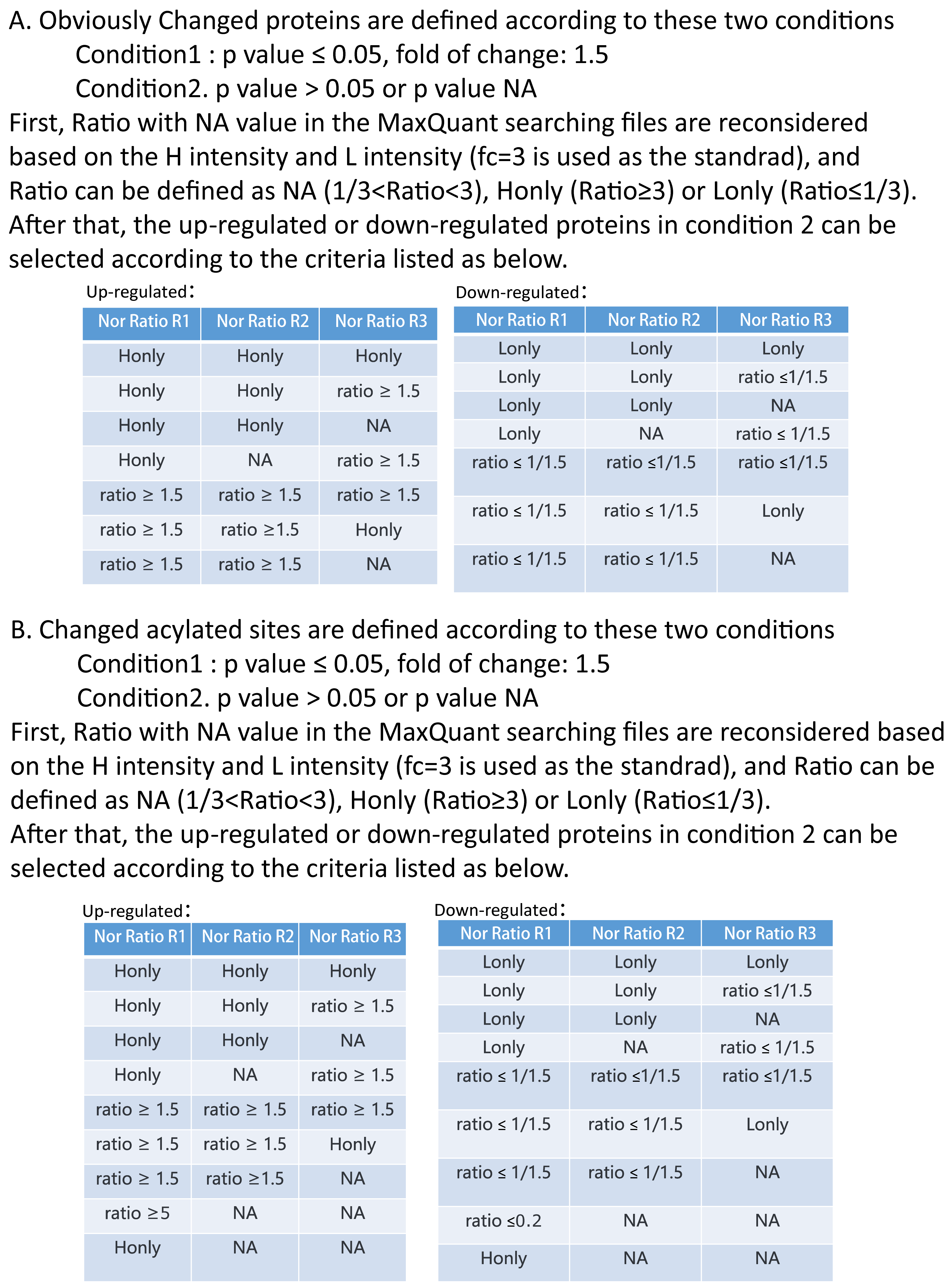

Supplement: FIG S1 [file mSystems.00424-19-sf001.tif]

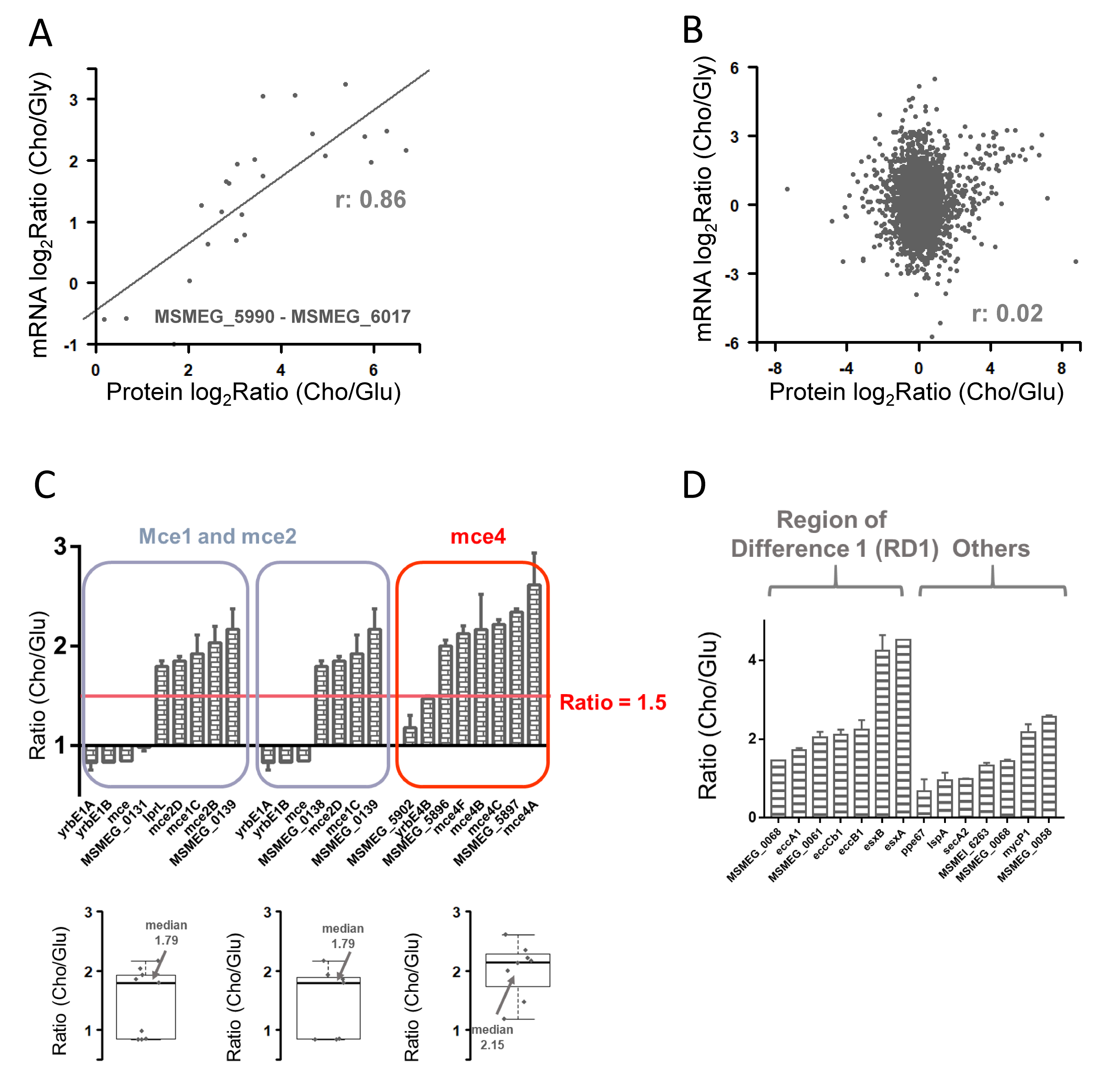

Supplement: FIG S2 [file mSystems.00424-19-sf002.tif]

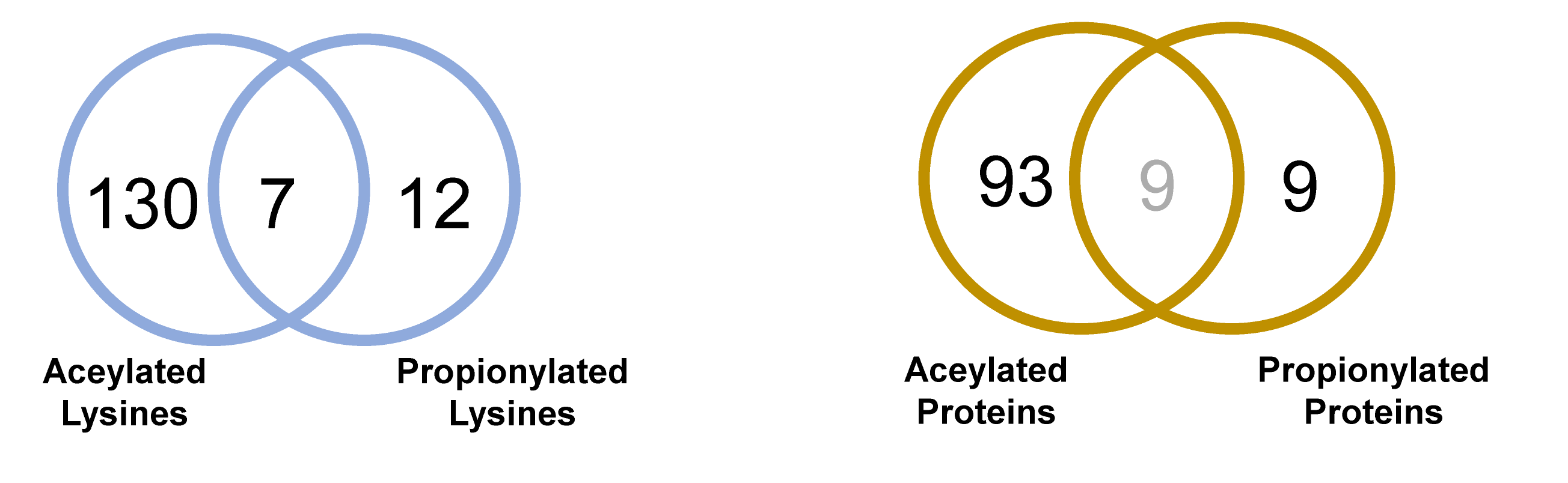

Supplement: FIG S3 [file mSystems.00424-19-sf003.tif]

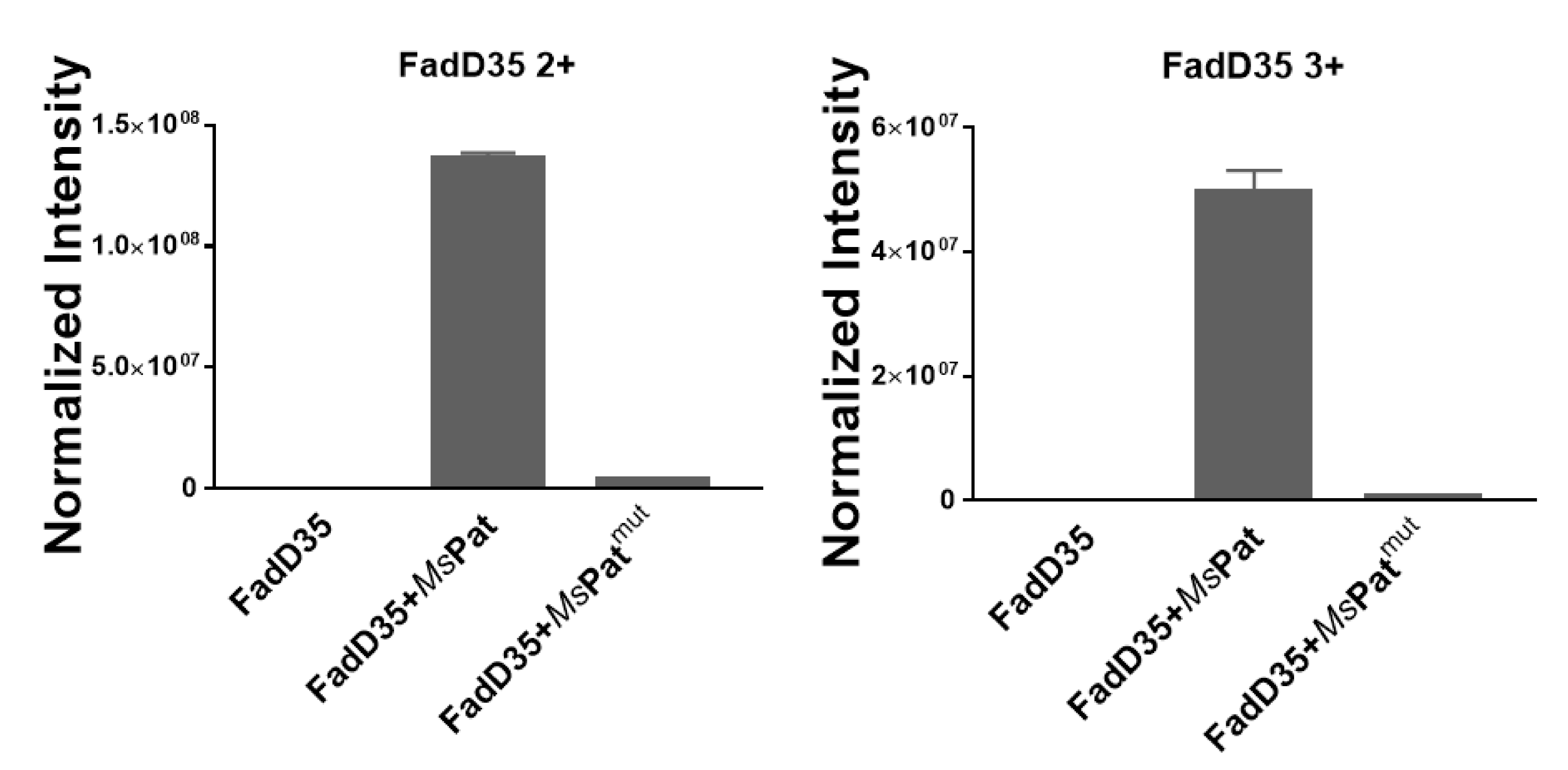

Supplement: FIG S4 [file mSystems.00424-19-sf004.tif]

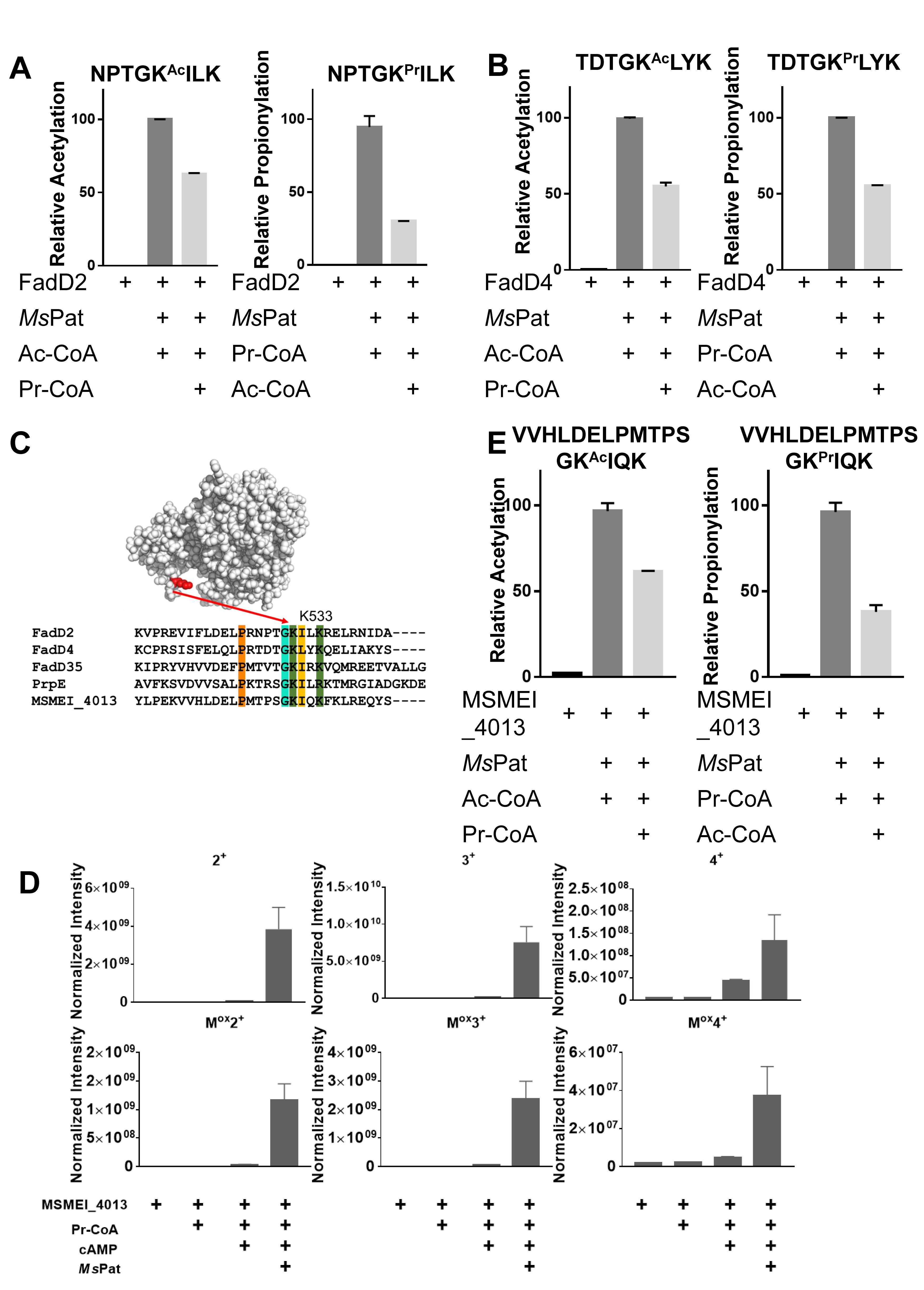

Supplement: FIG S5 [file mSystems.00424-19-sf005.tif]

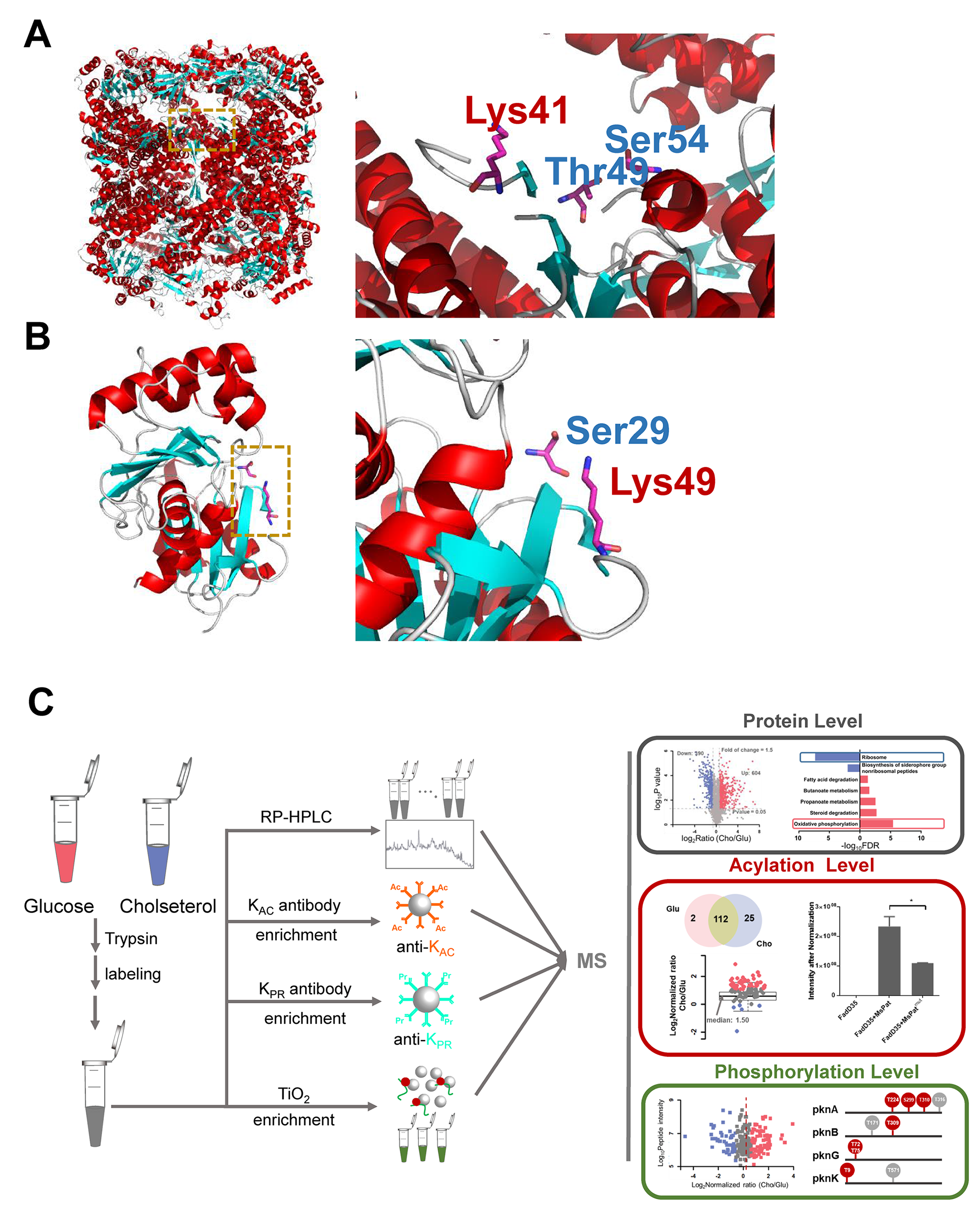

Supplement: FIG S6 [file mSystems.00424-19-sf006.tif]

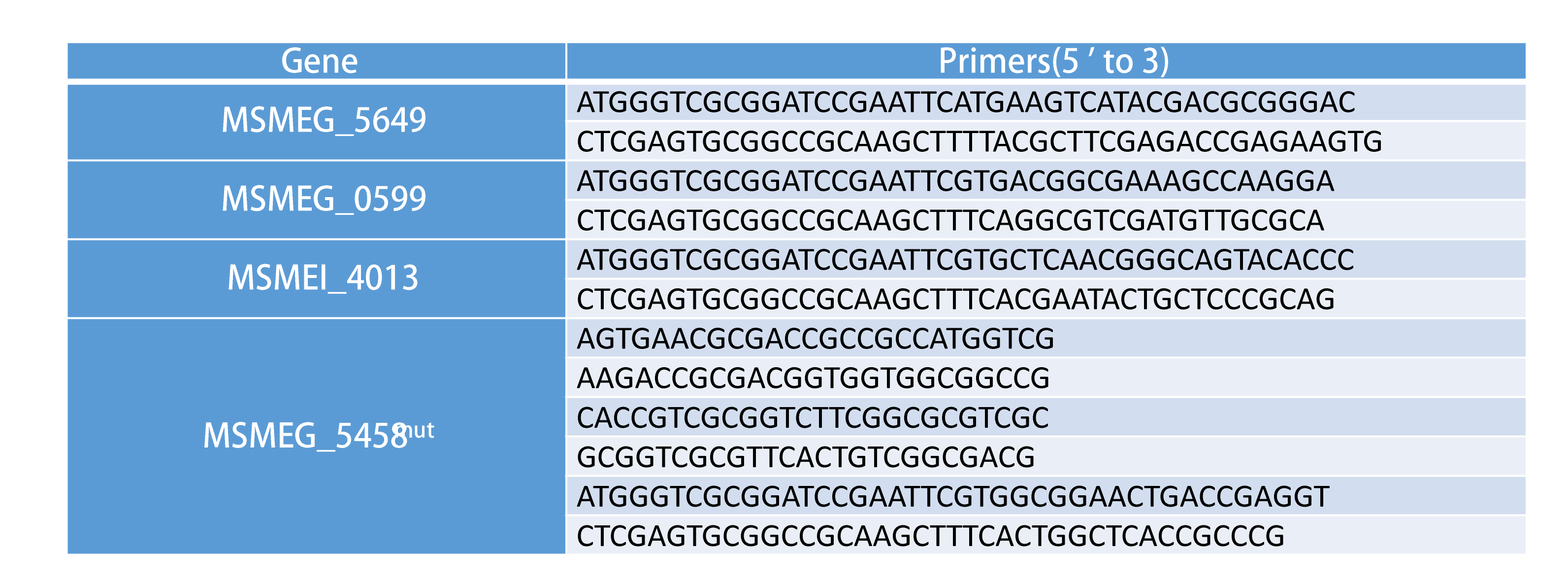

Supplement: FIG S7 [file mSystems.00424-19-sf007.tif]
